# Case Report: Drug-Induced Immune Haemolytic Anaemia Caused by Cefoperazone-Tazobactam/ Sulbactam Combination Therapy

**DOI:** 10.3389/fmed.2021.697192

**Published:** 2021-08-12

**Authors:** Yuanjun Wu, Yong Wu, Yanli Ji, Jiajie Liang, Ziyi He, Yanhui Liu, Li Tang, Ganping Guo

**Affiliations:** ^1^Department of Blood Transfusion, Dongguan Maternal and Child Health Hospital, Dongguan, China; ^2^Department of Blood Transfusion, Dongguan Tungwah Hospital, Dongguan, China; ^3^Institute of Clinical Blood Transfusion, Guangzhou Blood Center, Guangzhou, China; ^4^Dongguan Institute of Reproductive and Genetic Research, Dongguan Maternal and Child Health Hospital, Dongguan, China; ^5^Department of Transfusion Research, Dongguan Blood Center, Dongguan, China; ^6^Department of Research, Dongguan Maternal and Child Health Hospital, Dongguan, China

**Keywords:** cefoperazone, tazobactam, sulbactam, cefoperazone-dependent antibodies, nonimmunologic protein adsorption (NIPA), druginduced immune hemolytic anaemia (DIIHA), direct antiglobulin test (DAT)

## Abstract

There has previously been a report of a patient developing haemolytic anaemia following exposure to cefoperazone. Another case has been reported involving the detection of cefoperazone-dependent antibodies in the absence of immune haemolytic anaemia. To date, no serological evidence has been reported to suggest that cefoperazone can lead to drug-induced immune haemolytic anaemia (DIIHA). This report aims to fill these gaps in knowledge by describing a case of DIIHA caused by cefoperazone-dependent antibodies. A 59-year-old man developed fatal haemolytic anaemia while receiving cefoperazone-tazobactam or cefoperazone-sulbactam for the treatment of a lung infection that occurred after craniocerebral surgery. This eventually led to renal function impairment. Prior to the discontinuation of cefoperazone treatment, the patient showed strong positive (4+) results for both anti-IgG and anti-C3d direct antiglobulin test (DAT), while cefoperazone-dependent IgM and IgG antibodies were detected. The patient's plasma and O-type RBCs were incubated with tazobactam or sulbactam solution at 37°C for 3 h, the results of DAT for anti-IgG and anti-C3d were both positive. Forty-three days after the discontinuation of cefoperazone, the results of DAT for anti-IgG and anti-C3d were negative. Meanwhile incubation of the patient's fresh serum and his own RBCs with cefoperazone at 37°C, gave rise to mild haemolysis, and the results of DAT for both anti-IgG and anti-C3d were positive. It is suggested that cefoperazone-dependent antibodies can activate complement, and the non-immunologic protein adsorption effect of tazobactam or sulbactam can enhance IgG and complement binding to RBCs. This may promote the formation of immunocomplexes and complement activation, thereby aggravating haemolysis.

## Introduction

Drug-induced immune haemolytic anaemia (DIIHA) is believed to be caused by damage to red blood cells (RBCs), through drug-induced (drug-dependent and –independent) antibodies or non-immunologic protein adsorption (NIPA) (1–3). Almost all patients with DIIHA present severe anaemia caused by drug-induced antibodies (2). NIPA produces a positive direct antiglobulin test (DAT) result, and causes slow, covert, mild haemolysis (3–5). Approximately 140 drugs have been reported to cause DIIHA via drug-induced antibodies, and approximately 10 via NIPA (2–12).

Cefoperazone is a third-generation cephalosporin (13). Novaretti et al. reported that among 41 DAT-positive hospitalized patients with haematological disorders, serological tests found one to possess cefoperazone-dependent antibodies. However, this patient did not have any manifestations of immune haemolytic anaemia (14). Tazobactam and sulbactam are irreversible competitive β-lactamase inhibitors that can elucidate NIPA, leading to positive DAT results and elusive mild haemolysis (3, 4). Tazobactam or sulbactam used in combination with cephalosporins can improve the antibacterial effect; cefoperazone is commonly used in combination with tazobactam or sulbactam in the treatment of bacterial infections (15). Ling et al. reported that a 60-year-old woman, who had developed a lung infection following left atrial myxoma resection, developed haemolytic anaemia and tested positive in DAT for anti-C3d while being treated with cefoperazone and sulbactam (16). Cefoperazone-sulbactam combination therapy was thought to have induced DIIHA, but no serological tests were performed to detect drug-induced antibodies.

Here, we report a 59-year-old male who experienced severe haemolytic anaemia, along with liver and kidney dysfunction, while being administered an intravenous infusion of cefoperazone-tazobactam or cefoperazone-sulbactam for the treatment of pulmonary infection following cerebral haemorrhage and cerebral hernia surgery. Cefoperazone-dependent antibodies were detected in the plasma by serologic testing. To the best of our knowledge, this is the first reported case of DIIHA and renal failure caused by cefoperazone-dependent antibodies to be confirmed by serological methods. Combining the clinical evidence with immunological, biochemical and *in vitro* findings suggesting that the NIPA effect of tazobactam and sulbactam may promote the formation of immune complexes of cefoperazone-dependent antibodies with cefoperazone-coated RBCs *in vivo*; this would, in turn, activate the complement system, exacerbating haemolysis.

## Case Description

A 59-year-old male received cefoperazone-tazobactam (2:1) by intravenous infusion (2 g dose administered every 8 h; total 48 g cefoperazone-tazobactam administered through the course of treatment) for the treatment of a pulmonary infection and pleural effusion, which occurred following surgery for cerebral haemorrhage and cerebral hernia. Seven days after the start of treatment, yellowing of the patient's sclera was observed. On day 8, yellowing of the skin succeeded. Cefoperazone-tazobactam was discontinued and replaced with the more potent antibiotic, meropenem for the treatment of the severe pulmonary infection. The next day, therapeutic plasma exchange (TPE) was performed and bilirubin adsorption therapy was administered to address the aggravation of systemic jaundice and hyperbilirubinemia; bilirubin levels decreased significantly following this treatment. Four days later, the patient's haemoglobin (Hb) levels dropped to 61 g/L; a leukocyte-reduced red blood cells (LRBCs) infusion was administered, and the Hb levels rose to 103 g/L.

Treatment with meropenem for 12 days significantly alleviated the lung infection, and therapy was switched to the less potent cefoperazone-sulbactam (2:1) combination (3 g dose administered every 8 h; total 21 g cefoperazone-sulbactam administered through the course of treatment). TPE and bilirubin adsorption therapy were used again to address the reappearance of hyperbilirubinemia. Meropenem and levofloxacin were administered for 7 days.

On the 32nd day of hospitalization, the patient's condition improved and cefoperazone-sulbactam combination therapy was resumed (3 g dose administered every 8 h; total 24 g cefoperazone-sulbactam administered through the course of treatment). After 2 days, yellowing of the skin of the whole body was observed; after 3 days, antibiotic therapy was switched back to meropenem, and the yellow skin color faded.

Following this, computed tomography and pathological examinations revealed a malignant tumor in the left lobe of the patient's liver. On day 63 of hospitalization, the patient was subjected to superselective hepatic angiography and chemoembolization. After the operation, intravenous cefoperazone-sulbactam therapy was resumed at the same dose (total dose of 57 g administered during this stage of treatment). Three days after restarting cefoperazone-sulbactam administration, the patient's urine appeared brown, and the skin yellowish. The situation gradually aggravated. On day 4, the patient experienced dyspnoea, and metabolic acidosis; serum complement C3 and C4 levels decreased to 0.493 g/L (normal: 0.79–1.52 g/L) and 0.128 g/L (0.16–0.38 g/L), respectively. The patient was diagnosed with haemolytic crisis and transferred to the intensive care unit, where he was given blood volume supplementation, alkalized urine, dopamine, epinephrine and an intravenous infusion of methylprednisolone 1,000 mg. Hepatic and renal impairment occurred on day 5. Continuous renal replacement therapy was initiated on day 6. Blood exchange therapy was also administered. On day 8, TPE was performed and RBC replacement therapy was administered. Cefoperazone-sulbactam combination therapy was discontinued because of the suspected haemolytic association. On day 9, TPE was performed and LRBCs were infused. On day 10, washed red blood cells (WRBCs) were infused. The liver function, bilirubin and lactate dehydrogenase (LDH) then gradually returned to normal levels, and Hb stabilized at the baseline level. Blood urea nitrogen (BUN), creatinine levels, and urine output returned to near normal levels 45 days after cefoperazone-sulbactam was discontinued and continuous renal replacement therapy was then ceased.

**Figure 1:**
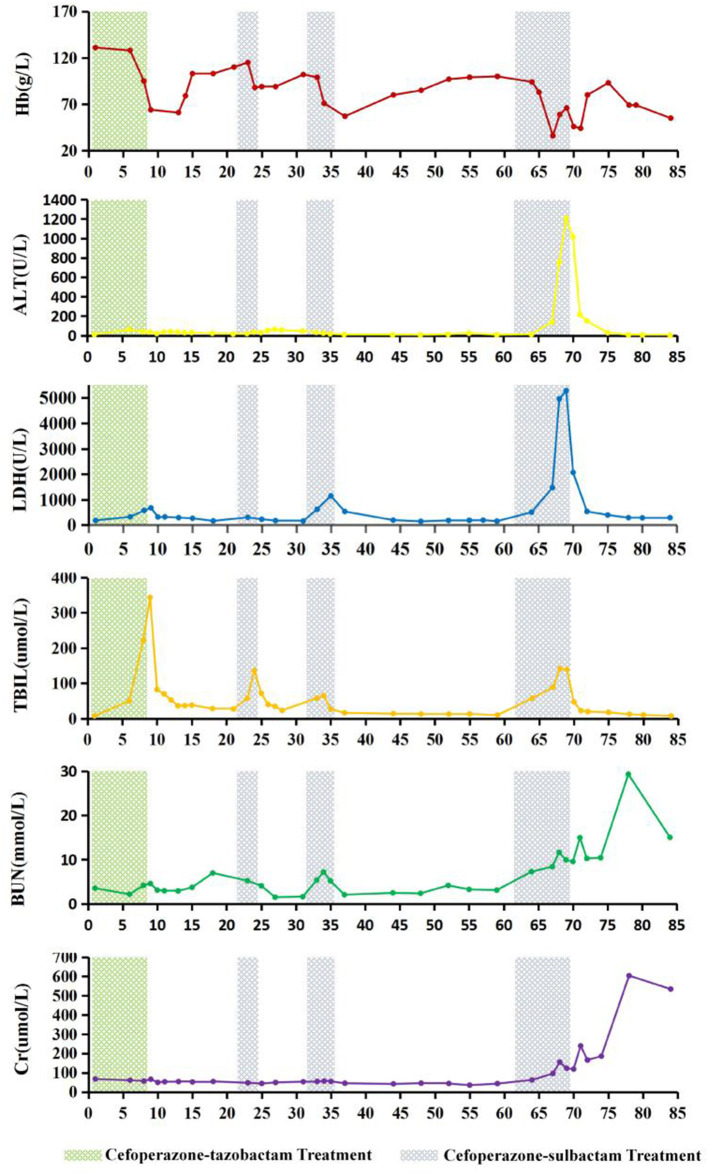
Changes in haemoglobin (Hb), alanine transaminase (ALT), lactate dehydrogenase (LDH), total bilirubin (TBIL), blood urea nitrogen (BUN), and creatinine (Cr) levels during cefoperazone-tazobactam and cefoperazone-sulbactam treatment. On the 9th day of hospitalization, therapeutic plasma exchange (TPE) with 2,100 mL of frozen plasma and bilirubin adsorption treatment were performed. On days 13 and 14, leukocyte-reduced red blood cells (LRBCs) prepared from 1,200 mL of whole blood were infused. On day 24, TPE with 2,000 mL of frozen plasma and bilirubin adsorption treatment were performed. On day 67 of hospitalization, 1,050 mL of frozen plasma and LRBCs prepared from 1,200 mL of whole blood were used for whole blood replacement therapy, and continuous renal replacement therapy was started. On day 69, TPE was performed using 4,100 mL of frozen plasma, followed by red blood cell exchange treatment with LRBCs prepared from 1,400 mL of whole blood. On day 70, TPE was repeated with 4,000 mL of frozen plasma, and LRBCs prepared from 400 mL of whole blood were infused. On the 71st day of hospitalization, washed red blood cells prepared from 800 mL of whole blood were infused.

Glucose-6-phosphate dehydrogenase (G6PD) activity and thalassemia gene screening results were found to be normal. On days 6 and 7 of the 4th course of cefoperazone therapy (during severe haemolysis), and the 6th day after cefoperazone was discontinued, the percentage of reticulocytes of the patient were 0.1, 0.3, and 4.7%, respectively (reference interval 0.5–1.5%). The dynamics of Hb, LDH, total bilirubin, alanine transaminase, BUN, creatinine, and cefoperazone-tazobactam and cefoperazone-sulbactam administration are shown in Figure 1. The patient had a history of aneurysm clipping, skull repair, and ventriculoperitoneal shunt placement, but no history of anaemia.

## Serological Test Results

As described by Leger and co-workers (17, 18), serological analysis including DAT, acid elution test, irregular RBC antibody screening were conducted on the patient's blood samples. Cefoperazone, tazobactam, sulbactam solutions, and the respective drug-coated RBCs were used to detect drug-dependent antibodies. *In vitro* experiments were conducted to verify the complement activation effect of drug-dependent antibodies, and the NIPA effect of tazobactam and sulbactam.

The results of irregular RBC antibody screening of plasma and acid eluates from the blood samples collected on days 67, 69 (cefoperazone treatment was discontinued), 71, 73, 75, 78, 85, 93, 104, 112, and 123 after hospitalization were all negative. DAT for anti-IgG and anti-C3d of the blood sample collected 2 days prior to the discontinuation of cefoperazone were strongly positive (4+), and gradually weakened for blood samples collected after cefoperazone discontinuation. The results of DAT for anti-IgG and anti-C3d became negative 43 and 16 days after the discontinuation of cefoperazone, respectively.

Cefoperazone solution (1–10 mg/mL) were incubated with O-type WRBCs and plasma collected on days 67, 69 (cefoperazone discontinued), 71, 73, 75, 78, 85, 93, 104, 112, and 123 after the patient's admission, cefoperazone-dependent antibodies were detected in every plasma sample, with the highest titre of 32. While the patient's plasma was incubated with cefoperazone-coated RBCs at 37°C for 1 h, cefoperazone-dependent antibodies were also detected, but the highest titre was only 1. Therefore, cefoperazone-coated RBCs are not suitable for the detection of cefoperazone-dependent antibodies. Tazobactam- or sulbactam-related drug-dependent antibodies were not detected in any of the blood samples. Details of drug-dependent antibody testing of the patient's blood sample collected on the 67th day of admission (2 days prior to the cessation of cefoperazone treatment) are shown in Table 1.

**Table 1:** Results of drug-dependent antibody tests of blood samples collected 2 days prior to the discontinuation of cefoperazone treatment.

**NO**.	**Reactive materials**										**Incubation conditions**	**Agglutination strength**	**DAT for anti-IgG**
	**P-P** ** (μL)**	**AE ** **(μL)**	**AB-P** ** (μL)**	**1 mg/mL CPZ** ** (μL)**	**1 mg/mL TBT** ** (μL)**	**1 mg/mL SBT** ** (μL)**	**UC-RBCs** ** (μL)**	**CPZ-RBCs** ** (μL)**	**TBT-RBCs** ** (μL)**	**SBT-RBCs** ** (μL)**			
1	100	/	/	100	/	/	50	/	/	/	37°C, 1 h	2+	4+
2	100	/	/	/	100	/	50	/	/	/	37°C, 1 h	–	–
3	100	/	/	/	/	100	50	/	/	/	37°C, 1 h	–	–
4	/	100	/	100	/	/	50	/	/	/	37°C, 1 h	–	–
5	/	/	100	100	/	/	50	/	/	/	37°C, 1 h	–	–
6	100	/	/	/	/	/	/	50	/	/	37°C, 1 h	–	1+
7	100	/	/	/	/	/	/	/	50	/	37°C, 1 h	–	–
8	100	/	/	/	/	/	/	/	/	50	37°C, 1 h	–	–
9	/	100	/	/	/	/	/	50	/	/	37°C, 1 h	–	–
10	/	/	100	/	/	/	/	50	/	/	37°C, 1 h	–	–

*P-P, patient's plasma; AE, acid eluate; AB-P, AB-type plasma with negative antibody screening test result; CPZ, cefoperazone; TBT, tazobactam; SBT, sulbactam; UC-RBCs, uncoated red blood cells; CPZ-RBCs, cefoperazone-coated red blood cells; TBT-RBCs, tazobactam-coated red blood cells; SBT-RBCs, sulbactam-coated red blood cells; DAT, direct antiglobulin test.*

*+, strong; –, negative*.

We treated the plasma with 2-mercaptoethanol (2ME) to cleave the IgM to identify the Ig type of cefoperazone-dependent antibodies. The patient's untreated plasma samples were incubated with 1 mg/mL cefoperazone and O-type RBCs at 37°C for 1 h; agglutination was observed by centrifugation in test tubes or in a Coombs test card. Plasma treated with 2ME was incubated with 1 mg/mL cefoperazone and O-type RBCs at 37°C for 1 h; no agglutination was observed by centrifugation in test tubes, but was observed in the Coombs test card. However, the titres of cefoperazone-dependent antibodies detected by the Coombs test card in untreated plasma and 2ME-treated plasma were similar. The results suggest that the patient's plasma contained both IgM and IgG cefoperazone-dependent antibodies, with the IgG type being predominant. Table 2 shows the changes in the DAT results and the titres of cefoperazone-dependent antibodies in the patient monitored over time.

**Table 2:** Results of direct antiglobulin tests and cefoperazone-dependent antibody titer tests.

**Hospitalization days** **(Days since discontinuation of cefoperazone)**	**DAT**		**1 mg/mL CPZ detected CPZ-dependent antibody titers with Coombs card**	
	**anti-IgG**	**anti-C3d**	**Plasma**	**2ME treated plasma**
67 (-2)	4	4	16	16
69 (0)	4	3	8	8
71 (2)	4	3	8	8
73 (4)	2	3	16	16
75 (6)	2	2	32	32
78 (9)	2	1	16	16
85 (16)	2	–	8	NT
93 (24)	2	–	8	NT
104 (35)	1	–	16	NT
112 (43)	–	–	16	NT
123 (54)	–	–	8	NT

*DAT, direct antiglobulin test; CPZ, cefoperazone; CPZ-RBCs, cefoperazone-coated red blood cells; 2ME, 2-mercaptoethanol; NT, not detected.*

*+, strong; –, negative*.

Plasma (with or without 2ME treatment) from blood samples collected 2 days prior to the discontinuation of cefoperazone-sulbactam treatment and O-type WRBCs was incubated with cefoperazone, tazobactam, or sulbactam (the final concentration of each drug was 1 mg/mL) at 37°C, to verify the NIPA effect of tazobactam or sulbactam whether promoted the binding of antibodies and complement to RBCs, and the results were certain. Detailed results of the *in vitro* control tests are shown in Table 3.

**Table 3:** *In vitro* validation of non-immunologic protein adsorption caused by tazobactam and sulbactam.

**NO**.	**Reactive materials**								**Incubation conditions**	**Agglutination or haemolysis**	**DAT**	
	**O-WRBCs** ** (μL)**	**P-P** ** (μL)**	**2ME PP** ** (μL)**	**AB-P** ** (μL)**	**PBS** ** (μL)**	**40 mg/mL CPZ** ** (μL)**	**40 mg/mL TBT** ** (μL)**	**40 mg/mL SBT** ** (μL)**			**anti-IgG**	**anti-C3d**
1	40	350	/	/	/	10	/	/	37°C, 3 h	3, NH	NT	NT
2	40	350	/	/	/	/	10	/	37°C, 3 h	NA, NH	4	±
3	40	350	/	/	/	/	/	10	37°C, 3 h	NA, NH	4	±
4	40	/	350	/	/	10	/	/	37°C, 3 h	NA, NH	4	1
5	40	/	350	/	/	/	10	/	37°C, 3 h	NA, NH	4	±
6	40	/	350	/	/	/	/	10	37°C, 3 h	NA, NH	4	±
7	40	350	/	/	10	/	/	/	37°C, 3 h	NA, NH	–	–
8	40	/	/	/	350	10	/	/	37°C, 3 h	NA, NH	–	–
9	40	/	/	350	/	10	/	/	37°C, 3 h	NA, NH	–	–
10	40	/	/	350	/	/	10	/	37°C, 3 h	NA, NH	4	–
11	40	/	/	350	/	/	/	10	37°C, 3 h	NA, NH	4	–

*O-WRBCs, O-type washed packed red blood cells with negative direct antiglobulin test results; P-P, patient's plasma; 2ME-PP, patient's plasma treated with 2-mercaptoethanol; AB-P, AB-type plasma with negative antibody screening test results; PBS, phosphate buffer solution (pH 7.2); CPZ, cefoperazone; TBT, tazobactam; SBT, sulbactam; DAT, direct antiglobulin test; NA, no agglutination; NH, no haemolysis; NT, not detected (the direct antiglobulin test could not be performed because it shows strong agglutination after centrifugation in the test tube).*

*+, strong; ±, slightly strong; –, negative*.

After the discontinuation of cefoperazone-sulbactam therapy for 43 days, the results of DAT for both anti-IgG and anti-C3d were negative, and the cefoperazone-dependent antibody titre was 16. The patient's fresh serum and his own RBCs were incubated with cefoperazone, tazobactam, or sulbactam solution at 37°C, to confirm that cefoperazone-dependent antibodies can cause complement activation. The results showed that cefoperazone-dependent antibodies can cause complement activation and mild haemolysis *in vitro*. Detailed results of the *in vitro* control test are shown in Table 4.

**Table 4:** *In vitro* testing of blood samples collected after negative direct antiglobulin test results for complement activation and non-immunologic protein adsorption.

**NO**.	**Reactive materials**						**Incubate at 37** ^****°****^ **C for 0.5 h**			**Incubate at 37** ^****°****^ **C for 1 h**			**Incubate at 37** ^****°****^ **C for 2 h**			**Incubate at 37** ^****°****^ **C for 3 h**		
	**P-WRBCs** ** (μL)**	**P-S** ** (μL)**	**AB-S** ** (μL)**	**40 mg/mL CPZ** ** (μL)**	**40 mg/mL TBT** ** (μL)**	**40 mg/mL SBT** ** (μL)**	**Agglutination or haemolysis**	**DAT for anti-IgG**	**DAT for anti-C3d**	**Agglutination or haemolysis**	**DAT for anti-IgG**	**DAT for anti-C3d**	**Agglutination or haemolysis**	**DAT for anti-IgG**	**DAT for anti-C3d**	**Agglutination or haemolysis**	**DAT for anti-IgG**	**DAT for anti-C3d**
1	40	350	/	10	/	/	WA^*^, NH	3+	–	WA^*^, WH	4+	±	WA^*^, WH	4+	1+	WA^*^, WH	4+	2+
2	40	350	/	/	10	/	NA, NH	±	–	NA, NH	+	–	NA, NH	3+	–	NA, NH	4+	–
3	40	350	/	/	/	10	NA, NH	±	–	NA, NH	+	–	NA, NH	3+	–	NA, NH	4+	–
4	40	/	350	10	/	/	NA, NH	–	–	NA, NH	–	–	NA, NH	–	–	NA, NH	–	–
5	40	/	350	/	10	/	NA, NH	±	–	NA, NH	+	–	NA, NH	3+	–	NA, NH	4+	–
6	40	/	350	/	/	10	NA, NH	±	–	NA, NH	+	–	NA, NH	3+	–	NA, NH	4+	–

*P-WRBCs, patient's washed and packed red blood cells; P-S, patient's fresh serum; AB-S, AB type healthy fresh human serum with negative antibody screening test results; CPZ, cefoperazone; TBT, tazobactam; SBT, sulbactam, Observe in tube, observe after centrifugation in a test tube; DAT, direct antiglobulin test; WA, Weak agglutination (dispersed after washing with pH 7.2 phosphate buffer solution), WH, weak haemolysis; NA, no agglutination; NH, no haemolysis.*

* *Before centrifugation, a portion of the red blood cells were aspirated and washed with pH 7.2 phosphate buffer solution; no agglutination observed under the microscope, so it could be used for DAT; +, strong; ±, slightly strong; –, negative*.

## Discussions

The patient developed severe haemolytic anaemia, hyperbilirubinemia, and elevated LDH levels during the cefoperazone-tazobactam/sulbactam combination therapy. The situation worsened and eventually progressed to haemolytic crisis and acute liver and kidney damage. Retrospective analysis revealed that the changes in the patient's Hb, LDH, total bilirubin, alanine transaminase, BUN, and creatinine levels were highly correlated with cefoperazone-tazobactam or cefoperazone-sulbactam administration. When the patient developed haemolytic crisis during the third course of cefoperazone-sulbactam therapy, the physician considered the possibility of DIIHA. At this time, DAT was performed using the patient's blood samples which were stored in the clinical laboratory for routine testing, and the results for anti-IgG and anti-C3d were strong positives (4+). However, the agglutination intensity of DAT for anti-IgG and anti-C3d gradually decreased after treatment with cefoperazone-sulbactam was discontinued, and were negative 43 and 16 days after the discontinuation of cefoperazone-sulbactam therapy, respectively. Irregular RBC antibody screening was repeatedly performed with the patient's plasma and acid eluate, and the results were negative. Cefoperazone-dependent antibodies were detected in the patient's plasma in the presence of cefoperazone solution; no drug-dependent antibodies were detected in the presence of tazobactam or sulbactam solution. Based on the patient's medical history and results of clinical, biochemical, and immunohaematological tests, we ruled out the possibility of haemolytic anaemia caused by G6PD deficiency (19, 20) and autoimmune haemolytic anaemia (21–25). The condition was diagnosed as DIIHA caused by cefoperazone-dependent antibodies and the NIPA effect of tazobactam and sulbactam.

Due to the lack of previous reports, the clinical features, immunohaematological characteristics, and treatment and prognosis of DIIHA caused by cefoperazone-dependent antibodies are poorly understood. Also due to the lack of vigilance to the risk of DIIHA caused by cefoperazone and the complexity of the patient's primary disease, haemolysis and jaundice during the repeated use of cefoperazone treatment were initially assumed to be an outcome of relapse of infection. Each time cefoperazone was discontinued and treatment was switched to the higher-level antibiotic meropenem, while the infection was effectively controlled, haemolysis and jaundice were also significantly relieved. This further misled the doctor's judgment, resulting in repeated incidences of cefoperazone-associated haemolysis and DIIHA not being diagnosed in time, ultimately resulting in liver and kidney dysfunction in the patient.

An increase in the reticulocyte count is a characteristic of haemolytic anaemia (23–26). Patients with special haemolytic anaemias, such as hyperhaemolysis syndrome, often exhibit reduced reticulocyte counts. This may be attributed to the potential destruction of reticulocytes during haemolysis. Macrophages in the endothelial system may play a key role in lysis of reticulocytes (27–29). In this patient, the percentage of reticulocytes was significantly below the reference level during the severe haemolysis that followed the fourth cycle of cefoperazone treatment. However, a significant increase in reticulocytes was detected 6 days after the discontinuation of cefoperazone treatment. However, among the reported cases of DIIHA, reticulocyte reduction is rare. The decrease in reticulocytes in this patient may have been a result of other underlying diseases, but it is not yet clear whether cefoperazone-dependent antibodies have a damaging effect on reticulocytes.

Drug-dependent antibodies can be detected by reacting with RBCs in a solution of related drug, or by reacting with drug-coated RBCs. The serological properties of drug-independent antibodies are similar to those of warm autoantibodies; they can be detected by the RBC antibody screening test without the need for related drug solutions or drug-coated RBCs (1, 2, 6, 18). Antibodies induced by different drugs may have different serological characteristics (2, 18). Our serological test showed that this patient did not produce drug-independent antibodies, but had produced cefoperazone-dependent IgM and IgG type antibodies. Cefoperazone-dependent antibodies were repeatedly tested within 43 days of discontinuation of cefoperazone. The range of antibody titres tested with 1 mg/mL cefoperazone solution were 8–32, while those tested with cefoperazone-coated RBCs were all only 1. Therefore, we found that cefoperazone solution, but not cefoperazone-coated RBCs, were suitable for the detection of cefoperazone-dependent antibodies.

The antibody titres that cause DIIHA are usually relatively high (2, 18, 30). Novaretti et al. previously reported that a patient with cefoperazone-dependent antibodies did not develop immune haemolytic anaemia (14). Therefore, it was assumed that cefoperazone (cefpirome) does not cause haemolytic anaemia (6). In this case, the highest titre of cefoperazone-dependent antibodies detected was 32, and very severe DIIHA occurred during treatment with cefoperazone-tazobactam or cefoperazone-sulbactam. The serum complements C3 and C4 were significantly reduced when the patient's haemolysis was most severe, but the DAT for anti-C3d showed a rare strong positive (4+) result. When the patient's plasma and O-type RBCs were incubated with cefoperazone solution at 37 °C, DAT for anti-C3d was positive. At 43 days after the discontinuation of cefoperazone, DAT for both anti-IgG and anti-C3d were negative, and the titre of cefoperazone-dependent antibodies was 16. When the patient's fresh serum and his own RBCs were incubated with cefoperazone solution at 37 °C, DAT for anti-C3d showed a positive (2+) result. The results show that cefoperazone-dependent antibodies exhibit strong complement activation. Novaretti et al. reported that a patient detected to have cefoperazone-dependent antibodies did not experience immune haemolytic anaemia (14); this may have been because the patient did not receive cefoperazone when the antibodies were detected.

When our patient had haemolysis, his plasma and O-type RBCs were incubated with tazobactam or sulbactam solution at 37°C, and the DAT results for anti-IgG and anti-C3d were positive, suggesting that the NIPA effect of tazobactam or sulbactam may promote the binding of IgG and complements to RBCs. However, NIPA alone does not cause serious DIIHA (3–5). Therefore, while the patient was receiving cefoperazone-tazobactam or cefoperazone-sulbactam, the NIPA effect of tazobactam or sulbactam may have promoted the specific binding of cefoperazone-dependent antibodies with the RBCs coated with cefoperazone in the patient's body, promoting the formation of immune complexes and complement activation. This eventually led to the aggravation of haemolysis.

There is little evidence for the appropriate course of treatment for DIIHA. It is very important to discontinue the administration of the related drugs in time and provide the right targeted treatment (31). Corticosteroids are widely used in the treatment of DIIHA, but their effectiveness is difficult to be distinguished from the benefits of stopping the use of the causative drug (8, 32). Rituximab, azathioprine, cyclophosphamide, cyclosporine, danazol, mycophenolate, and IV immunoglobulin are considered effective in the treatment of severe or refractory autoimmune haemolytic anaemia (23, 33), but their efficacy in the treatment of DIIHA remains unclear (8). It has been reported that the administration of TPE to patients with severe DIIHA and renal impairment can be effective (8). Our patient experienced recurring haemolytic anaemias associated with cefoperazone-tazobactam or cefoperazone-sulbactam, due to delay in diagnosis. During the period, TPE, bilirubin adsorption, blood transfusion, and whole blood replacement were given several times for the treatment of anaemia, liver damage and hyperbilirubinemia. Continuous renal replacement therapy was administered after the occurrence of renal impairment. Clinical monitoring and laboratory test results suggested that these symptomatic treatments were effective. But there was a lack of knowledge about the condition, leading to a delay in diagnosis. Therefore, there is an urgent need to establish guidelines for the diagnosis and treatment of DIIHA.

## Data Availability Statement

The original contributions presented in the study are included in the article/Supplementary Material. Further inquiries can be directed to the corresponding author.

## Ethics Statement

The studies involving human participants were reviewed and approved by Ethical Committee of Dongguan Maternal and Child Health Hospital. The patients/participants provided their written informed consent to participate in this study.

## Author Contributions

YJW, YW, YLJ, and JJL contributed to the conception and design of the study and data analysis. YJW, JJL, ZYH, YHL, and LT prepared the draught and final manuscript. YJW, YW, YLJ, and GPG performed the immunohematological test. All authors listed have made a contribution to the work, and approved it for publication.

## Conflict of Interest

The authors declare that the research was conducted in the absence of any commercial or financial relationships that could be construed as a potential conflict of interest.

## Publisher's Note

All claims expressed in this article are solely those of the authors and do not necessarily represent those of their affiliated organizations, or those of the publisher, the editors and the reviewers. Any product that may be evaluated in this article, or claim that may be made by its manufacturer, is not guaranteed or endorsed by the publisher.

## Abbreviations

DIIHA: drug-induced immune haemolytic anaemia
RBC: red blood cell
RBCs: red blood cells
NIPA: non-immunologic protein adsorption
DAT: direct antiglobulin test
TPE: therapeutic plasma exchange
Hb: haemoglobin
LRBCs: leukocyte-reduced red blood cells
WRBCs: washed red blood cells
LDH: lactate dehydrogenase
BUN: blood urea nitrogen
G6PD: Glucose-6-phosphate dehydrogenase
PBS: phosphate buffer solution
2ME: 2-mercaptoethanol.
